# *Salmonella* spp acute enterocolitis - an overview of antibiotic therapy and chemosensitivity patterns among children

**DOI:** 10.1186/1471-2334-13-S1-P37

**Published:** 2013-12-16

**Authors:** Gheorghiță Jugulete, Monica Luminos, Anca Drăgănescu, Angelica Vişan, Mădălina Maria Merişescu, Anuța Bilaşco, Sabina Șchiopu, Endis Osman, Olga Dorobăț

**Affiliations:** 1 National Institute for Infectious Diseases “Prof. Dr. Matei Balş”, Bucharest, Romania; 2 Carol Davila University of Medicine and Pharmacy, Bucharest, Romania

## Background

Acute *Salmonella* spp infections represent an important public health issue worldwide, particularly among children. Romania is a country with endemic *Salmonella* infections which reports occasionally summer outbreaks.

Our study aimed to analyze the submitted cases and to appreciate the germ’s susceptibility to antibiotics, starting from the urging issue related to the emerging resistance of *Enterobacteriaceae* to common drugs.

## Methods

We conducted a clinic-based retrospective (2010-2013) surveillance that analyzed the confirmed pediatric cases of *Salmonella* spp infections treated at the National Institute for Infectious Diseases “Prof. Dr. Matei Balş”, Bucharest, Romania. The clinical and demographic patient features followed were: age, sex, home environment, severity of the disease and the complications. All the bacterial strains were isolated after culturing the stool samples on differential culture media and the identification was performed respecting the standard laboratory methodology. The antibiotic sensitivity spectrum was determined using the API ATB G-5 tests for the following drugs: ampicillin, amoxicillin/clavulanate, ampicillin/sulbactam, trimethoprim/sulfamethoxazole, nalidixic acid, fluoroquinolones, tetracycline and third generation cephalosporins.

## Results

During the studied period we identified 135 cases of acute *Salmonella* spp infection among hospitalized children, which stands for 8.5% of the total cases of acute diarrheal diseases. The most affected age group was the 1-4 years group, with a male predominance. The serotype distribution was: AO – 12.6%, BO – 31.1%, CO – 19.2% and DO – 37.1%. The chemosensitivity analysis revealed: 100% sensitivity to fluoroquinolones, carbapenems and third generation cephalosporins, 92.6% sensitivity to nalidixic acid and trimethoprim/sulfamethoxazole, 72.6% sensitivity to amoxicillin/clavulanate, ampicillin/sulbactam, 62.9% sensitivity to tetracycline and only 53.3% sensitivity to ampicillin.

## Conclusions

*Salmonella* spp induced enterocolitis represents a major morbidity cause among children, especially during summers. The chemosensitivity analysis revealed excellent sensitivity of the strains to fluoroquinolones, carbapenems, third generation cephalosporins and nalidixic acid, good sensitivity of the strains to trimethoprim/sulfamethoxazole, amoxicillin/clavulanate and ampicillin/sulbactam and weak sensitivity to tetracycline and ampicillin. The subgroups analysis showed the increasing number of resistant strains in consecutive years, fact that highlights the importance of correct antibiotic prescription.

